# La trochantérite tuberculeuse, un diagnostic souvent difficile

**DOI:** 10.11604/pamj.2016.23.111.9189

**Published:** 2016-03-16

**Authors:** Younes Mhammdi, Moncef Boufettal, Mohamed Kharmaz, Abdou Lahlou, Mustapha Mahfoud, Ahmed EL Bardouni, Mohamed Saleh Berrada

**Affiliations:** 1 Service de Traumatologie Orthopédie, Hopital Ibn Sina, CHU Rabat, Maroc

**Keywords:** Tuberculose, trochantérite, abcès, Tuberculosis, trochanteritis, abscess

## Abstract

La trochantérite tuberculeuse est une affection rare (<2% des tuberculoses ostéo-articulaires), même en pays à forte endémie, elle est caractérisée par une symptomatologie insidieuse rendant souvent son diagnostic tardif. Les auteurs rapportent une série de 9 cas, le diagnostic a été posé sur des études bactériologiques et histologiques après un bilan d'imagerie (échographie, IRM, TDM). Le traitement de cette affection est médical (antibiothérapie antituberculeuse), et la chirurgie ne trouve sa place qu'en cas de complications et permet d'améliorer l’évolution.

## Abstract (en)

Trochanteric tuberculosis is a rare disease (<2% of osteoarticular tuberculosis), even in high-endemic countries. It is characterized by insidious symptoms. For this reason, diagnosis is often delayed. The authors report a series of 9 cases, the diagnosis was made by bacteriological and histological examinations after imaging assessment (ultrasound, MRI, CT scan). The treatment of this disease is medical (anti-tuberculous antibiotic). Surgical treatment is usually reserved for complications and improves disease progression

## Introduction

La trochantérite tuberculeuse est due à la localisation du bacille de Koch au niveau du grand trochanter et/ou dans sa bourse. C'est une entité rare et ne représente que 2% des tuberculoses ostéo-articulaires, et 21% des ostéites tuberculeuses [1, 2]. Son diagnostic reste difficile et tardif car cette ostéite bacillaire a une symptomatologie discrète et une évolution longue. Les nouvelles techniques d'imageries permettent actuellement un diagnostic plus précoce, la confirmation du diagnostic se fait toujours sur l’étude histologique.

## Méthodes

Il s'agit d'une étude rétrospective de neuf cas de trochantérite tuberculeuse colligés au service de traumatologie orthopédie du centre hospitalier universitaire Ibn Sina de Rabat entre 2004 et 2011. La recherche d'un contage tuberculeux ou d'antécédents de tuberculose pulmonaire était systématique dans notre bilan clinique. L'examen physique recherchait une tuméfaction, des fistules, une douleur provoquée au niveau du grand trochanter, et évaluait également l’état de mobilité de la hanche. L'examen général recherchait d'autres localisations tuberculeuses en particuliers pulmonaire et rachidienne. Des bilans biologiques bactériologiques et surtout radiologique principalement l'IRM ont permis de suspecter le diagnostic. L’étude histologique de la paroi de l'abcès froid avait confirmé le diagnostic de tuberculose caséo-folliculaire dans tous les cas. Sur le plan thérapeutique, Le traitement médical par les antituberculeux a été prescrit pour l'ensemble de nos malades pour une durée de douze mois sous l’égide d'un pneumophtisiologue. Le traitement chirurgical réalisé huit fois, a consisté à évacuer l'abcès froid, à réséquer sa capsule, et à cureter le grand trochanter. Dans un seul cas, on s'est juste contenté de réaliser des ponctions à répétition.

## Résultats

**Figure 1:**
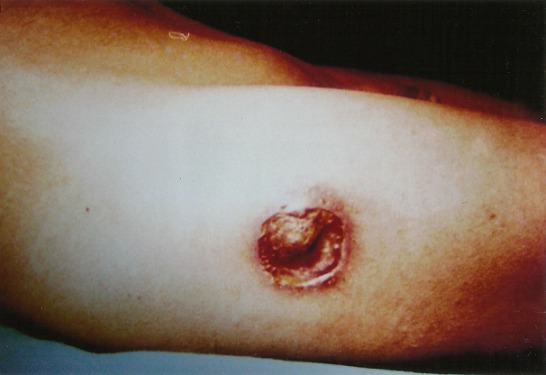
Fistule à la face externe de la cuisse

La série comportait six femmes et trois hommes. L’âge moyen était de 51 ans (25- 70 ans). Le délai diagnostic était en moyenne de 10 mois (5 à 20 mois) après le début de la symptomatologie. Une patiente présentait dans ses antécédents une tuberculose pulmonaire et une autre présentait un mal de Pott cervical concomitant. Tous les patients ont consulté pour une douleur mécanique modérée en regard du grand trochanter qui était associée à une tuméfaction dans huit cas et à une fistule cutanée dans trois cas. La tuméfaction siégeait six fois au niveau de la cuisse et deux fois à la fesse, il s'agissait à chaque fois d'une masse rénitente, dépressible sans signes inflammatoires cutanés qui avait fi'stulisé à la peau dans trois cas, deux fois au niveau de la cuisse et une fois en regard du grand trochanter (Figure 1). La mobilité de la hanche atteinte était normale chez tous les patients. L’état général était conservé sauf la patiente avec mal de Pott cervical.

**Figure 2:**
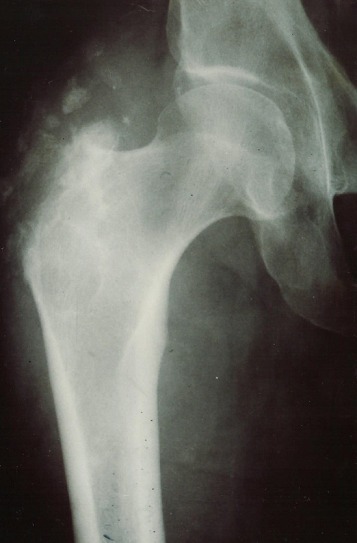
Image radiographique des remaniements du grand trochanter

**Figure 3:**
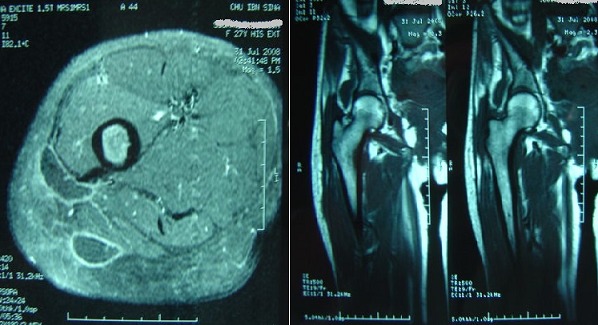
IRM montrant une collection en contact du grand trochanter

Sur le plan radiologique, huit fois les radiographies conventionnelles montraient des remaniements du grand trochanter à type d'irrégularités de son bord externe et des lacunes entourées de condensation de son corps ainsi que des calci'cations des parties molles adjacentes (Figure 2), La tomodensitométrietrie (TDM) réalisée chez quatre patients révélait des lésions trochantériennes identiques et des abcès des partie molles sous forme d'images de densité hydrique dont la paroi prenait le produit de contraste. L’échographie mettait en évidence une masse anéchogène. Ces abcès étaient localisés six fois au niveau de la cuisse et deux fois au niveau de la région fessière (Figure 2). L'IRM réalisée chez six de nos patients, montrait l'abcès en hypo signal en T1 et en hyper signal régulier en T2; elle a permis de mieux étudier l'extension des abcès, leurs rapports vasculo-nerveux et l’état de la hanche (Figure 3).

Le bilan biologique était normal sauf la VS qui était trois fois modérément élevée et trois fois très élevée et associée à une hyperleucocytose dans les cas de surinfection par le staphylocoque pathogènene. L'IDR à la tuberculine était très positive dans trois cas. La recherche de BK à l'examen direct et à la culture n’était positive que dans trois cas. Trois fois il y'avait une surinfection par le staphylocoque doré pathogène. La preuve anatomo-pathologique de la tuberculose a été apportée par la biopsie. Elle a révélé une tuberculose caséo- folliculaire dans tous les cas. Avec un recul moyen de trois ans, il y'avait une indolence totale et une mobilité normale de la hanche avec cicatrisation des fistules cutanées. Jusqu'au dernier contrôle, il n'y avait pas de récidive, La radiographie de la hanche de contrôle montrait une amputation partielle du grand trochanter sans atteinte de l'articulation coxo-fémorale.

## Discussion

La trochantérite tuberculeuse est rare, même dans les pays à forte endémie tuberculeuse et ne représente que 0,2 à 2% de l'ensemble des foyers tuberculeux ostéo-articulaires [3, 4] Elle tient son intérêt dans sa localisation péri-articulaire et du risque d'envahissement secondaire de la hanche. Sa pathogénie est controversée, le bacille de Koch est d'origine pulmonaire dans 97% des cas et arrive par voie hématogène.

Le diagnostic de la trochantérite tuberculeuse est souvent tardif et longtemps méconnu car l’évolution est lente et la symptomatologie est discrète et non spécifique [2, 5]. La symptomatologie est dominée par une douleur chronique modérée de la région trochantérienne et surtout une tuméfaction de la face externe de la cuisse ou plus rarement de la fesse due à l'abcès froid migrateur qui suit les gaines musculaires [5]. Le bilan biologique est non spécifique, la vitesse de sédimentation (VS) est modérément augmentée dans la trochantérite tuberculeuse isolée, elle est élevée et associée à une hyperleucocytose en cas de surinfection. Sur le plan radiologique, la radiographie standard de la hanche peut être normale ou peut montrer une ostéoporose du grand trochanter [6]; Une lyse osseuse complète du grand trochanter est rarement rapportée [7]. Dans les cas évolués de notre série, il y'a des remaniements trochantériens à type d’érosions, géodes, lacunes, condensation et séquestres. Les calcifications des parties molles péri-trochantériennes sont inconstantes. L’échographie de la hanche, de la fesse et surtout de la cuisse peut visualiser des abcès superficiels sous forme d'images hypo ou anéchogènes [6] et permet de guider leur ponction [8]. La tomodensitométrie permet une mise en évidence de l'abcès sous forme d'une image de densité hydrique dont la paroi prend les produit de contraste, elle fournit des informations morphologiques intéressantes sur les collections (siège, taille, nombre) [6, 8, 9]. L'imagerie par résonance magnétique permet de détecter les modifications de la moelle osseuse et met en évidence la bursite [4]. Elle permet par ailleurs, en analysant les parties molles périphériques, de reconnaître l'abcès s'il est de petite dimension et d’étudier son prolongement quand il est volumineux [10]. L'IRM reste donc plus performante que la TDM, permettant d’établir un bilan local plus précis de cette pathologie, elle constitue pour la plupart des auteurs l'examen radiologique de choix. L’étude anatomo-pathologique permet de confirmer le diagnostic de certitude contrairement à l’étude bactériologique qui n'est pas souvent concluante.

Le traitement optimal de la trochanterite tuberculeuse reste controversé. Pour réduire le risque de diffusion mycobacérienne chez les personnes avec atteinte du trochanter, une antibiothérapie antituberculeuse doit être lancée immédiatement une fois le diagnostic définitif est confirmé [11]. Cette thérapie anti bacillaire seule peut éradiquer la maladie à tout moment de l’évolution et sa durée est de 12 mois, la chirurgie est indiquée en présence de complications [12], elle permet accélérer la guérison des cas évolués et d'eviter les récidives [13]. Une Réactivation de la maladie après thérapie anti bacillaire associée ou non à un traitement chirurgical a été reportée. [14, 15] Un suivi régulier est donc indispensable.

## Conclusion

Le diagnostic de la trochantérite tuberculeuse est tardif, chose confirmée par notre étude où tous les patients ont consultés au stade de complications, ceci est due à une symptomatologie insidieuse et tolérée, il faut y penser devant toute douleur de la région trochantériernne associée ou non à une tuméfaction de la cuisse avec ou sans fistules cutanées, L'avènement de moyens diagnostiques modernes (TDM et IRM) permet la possibilité d'un diagnostic au début de la maladie permettant ainsi un traitement précoce et des meilleurs résultats. Les bilans biologiques et bactériologiques sont non spécifiques, c'est l’étude histologie qui a permis de poser le diagnostic pour tous nos patients. Le traitement est surtout médical à base d'antibacillaires, le traitement chirurgical trouve son intérêt dans les cas évolués et permet de raccourcir l’évolution.

### Etat des connaissance sur le sujet

- Pathologie rare
- Diagnostic souvent tardif
- Le plus souvent diagnostic histologique au stade de complications

### Contribution de notre étude a la connaissance

- La non spécificité des examens biologiques et bactériologiques dans la pose du diagnostic
- La supériorité de l'IRM et son aide précieuse permettant un diagnostic précoce
- L'intérêt du traitement chirurgical
